# Correction: Li et al. Zanubrutinib Ameliorates Cardiac Fibrosis and Inflammation Induced by Chronic Sympathetic Activation. *Molecules* 2023, *28*, 6035

**DOI:** 10.3390/molecules31101561

**Published:** 2026-05-08

**Authors:** Wenqi Li, Shuwen Zhu, Jing Liu, Zhigang Liu, Honggang Zhou, Qianyi Zhang, Yue Yang, Li Chen, Xiaowei Guo, Tiantian Zhang, Lingxin Meng, Dan Chai, Guodong Tang, Xiaohe Li, Cheng Yang

**Affiliations:** 1 State Key Laboratory of Medicinal Chemical Biology, College of Pharmacy and Tianjin Key Laboratory of Molecular Drug Research, Nankai University, Haihe Education Park, 38 Tongyan Road, Tianjin 300353, China; 1120210653@mail.nankai.edu.cn (W.L.); 2120211341@mail.nankai.edu.cn (S.Z.); 2120221626@mail.nankai.edu.cn (J.L.); 1120220741@mail.nankai.edu.cn (Z.L.); honggang.zhou@nankai.edu.cn (H.Z.); zqy15027605203@163.com (Q.Z.); 15502617145@163.com (Y.Y.); chenliesther0620@163.com (L.C.); gxw990829@163.com (X.G.); zhangtt_gala@163.com (T.Z.); 13356720390@163.com (L.M.); c2094760933@163.com (D.C.); 2 Tianjin International Joint Academy of Biomedicine, Tianjin 300457, China; 3 Department of Cardiology, Beijing Hospital, National Center of Gerontology, Institute of Geriatric Medicine, Chinese Academy of Medical Sciences, Beijing 100730, China

In the original publication [1], there was a mistake in Figure 1. The authors noticed an error in Figure 1b when they reviewed this published work. The incorrect image was used for the ISO + Met group. The corrected Figure 1 appears below. The authors state that the scientific conclusions are unaffected. This correction was approved by the Academic Editor. The original publication has also been updated.

## Figures and Tables

**Figure 1:**
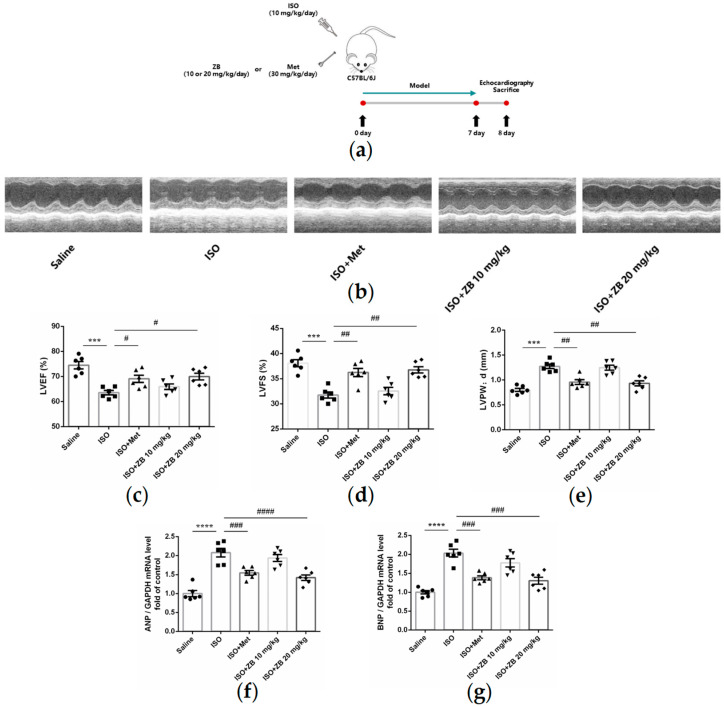
ZB prevents ISO-induced cardiac dysfunction. (**a**) Dosing regimen in ISO-induced cardiac fibrosis model. Mice were treated by daily subcutaneous injection of 10 mg kg^−1^ d^−1^ ISO for 7 days. For 7 days, 10 mg/kg, 20 mg/kg ZB, 30 mg/kg Met or equal volumes of Saline was gavaged daily as indicated. Met was used as a positive control; (**b**) Representative echocardiographic M-mode images of left ventricles from mice at day 8; (**c**) Echocardiographic measurement of LVEF (*n* = 6); (**d**) Echocardiographic measurement of LVFS (*n* = 6); (**e**) Quantitative analysis of LVPW;d (*n* = 6); (**f**) The mRNA levels of ANP in heart tissues (*n* = 6); (**g**) The mRNA levels of BNP in heart tissues (*n* = 6); Quantification of ANP and BNP were normalized to GAPDH. The data are shown as mean ± SEM (one-way ANOVA with Tukey’s post-hoc multiple comparison tests). ***, *p* < 0.001, ****, *p* < 0.0001 vs. Saline; #, *p* < 0.05, ##, *p* < 0.01, ###, *p* < 0.001, ####, *p* < 0.0001 vs. ISO. ISO, isoproterenol; Met, metoprolol; ZB, Zanubrutinib; LVEF, left ventricular ejection fraction; LVFS, left ventricular fractional shortening; LVPW;d, diastolic left ventricular posterior wall thickness.

## References

[1] Li W., Zhu S., Liu J., Liu Z., Zhou H., Zhang Q., Yang Y., Chen L., Guo X., Zhang T. (2023). Zanubrutinib Ameliorates Cardiac Fibrosis and Inflammation Induced by Chronic Sympathetic Activation. Molecules.

