# Supplementary material for: Zanubrutinib Ameliorates Cardiac Fibrosis and Inflammation Induced by Chronic Sympathetic Activation
Source: Molecules. 2023 Aug 12;28(16):6035. doi: 10.3390/molecules28166035 (PMC10458081; doi:10.3390/molecules28166035)
Supplement: Supplementary file 1 [file molecules-28-06035-s001.zip › molecules-2458338-supplementary.pdf]

Supplementary Material

Zanubrutinib Ameliorates Cardiac Fibrosis and Inflammation Induced by Chronic Sympathetic Activation

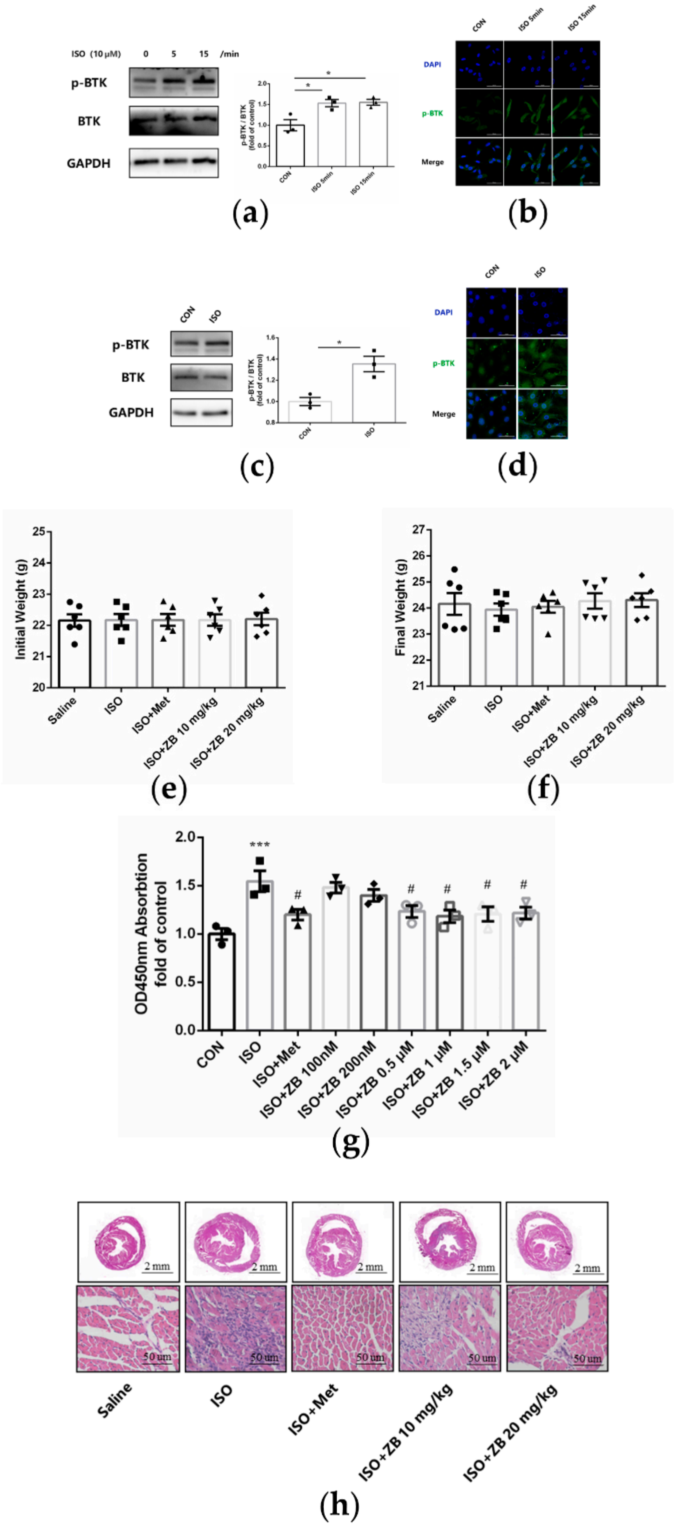

Supplemental Figure S1. Phospholyation of BTK with short term ISO treated cardiac fibroblasts. (**a**) Western Blot analysis for phospho-BTK level after 5 and 15 min ISO (10 μmol L^{-1}) treatment in NRCFs (n=3); (**b**) Immunofluorescence staining of phospho-BTK after 5 and 15 min ISO (10 μmol L^{-1}) treatment in NRCFs. Scale bar: 50 μm. NRCFs were starved for 24 h, then treated with ISO (10 μmol L^{-1}) for 5 min and 15min. Phospholyation of BTK with long term ISO treated cardiac fibroblasts. (**c**) Western Blot analysis for phospho-BTK level after 24 h ISO (10 μmol L^{-1}) treatment in NRCFs (n=3); (**d**) Immunofluorescence staining of phospho-BTK after 24 h ISO (10 μmol L^{-1}) treatment in NRCFs. Scale bar: 50 μm. NRCFs were starved for 24 h, then treated with ISO (10 μmol L^{-1}) for 24 h. Mice weight of each group. (**e**) Initial body weight of animal experiment from each group (n=6); (**f**) Final body weight of animal experiment from each group (n=6). Cell viability in different doses of ZB in cardiac fibroblasts. (**g**) Analysis of cell viability in NRCFs by CCK-8. NRCFs were starved for 24 h, incubated with ZB (0.1-2 μmol L^{-1}) or equal DMSO for 1 h and then treated with ISO (10 μmol L^{-1}) for 24 h. Met was used to be a positive control. H&E staining in heart tissue in cardiac inflammation model. (**h**) Representative 1× and 40× images and quantification of H&E staining in heart tissue (n=6). Scale bar (upper): 2 mm; scale bar (lower): 50 μm. The data are shown as mean±SEM (one-way ANOVA with Tukey's post-hoc multiple comparison tests).
